# Validation of Clinical Treatment Score post-5 years (CTS5) risk stratification in premenopausal breast cancer patients and Ki-67 labelling index

**DOI:** 10.1038/s41598-020-74055-3

**Published:** 2020-10-08

**Authors:** Janghee Lee, Chihwan Cha, Sung Gwe Ahn, Dooreh Kim, Soeun Park, Soong June Bae, Jeeye Kim, Hyung Seok Park, Seho Park, Seung Il Kim, Byeong-Woo Park, Joon Jeong

**Affiliations:** 1 Department of Surgery, Gangnam Severance Hospital, Yonsei University College of Medicine, 712 Eonjuro, Gangnam-gu, Seoul, 06273 Republic of Korea; 2 Department of Surgery, Severance Hospital, Yonsei University College of Medicine, Seoul, Republic of Korea; 3 Department of Surgery, Sacred Heart Hospital, Hallym University, Dongtan, Republic of Korea; 4 Department of Surgery, Hanyang University College of Medicine, Seoul, Republic of Korea

**Keywords:** Cancer, Oncology

## Abstract

This study aimed to validate the Clinical Treatment Score post-5 years (CTS5)-based risk stratification in a cohort comprising pre- and postmenopausal patients with estrogen receptor (ER)–positive breast cancer. We investigated the clinicopathologic parameters including Ki-67 labelling index (LI) to identify factors affecting late distant recurrence (DR). Women with ER-positive breast cancer who were free of DR for 5 years were identified between January 2004 and December 2009. We investigated the risk of late DR (5–10 years) according to the CTS5 risk group. Cox regression analysis was used to determine the prognostic performance of CTS5 and identify factors associated with late DR. In all, 680 women were included. Of these, 379 (55.7%) were premenopausal and 301 (44.3%) were postmenopausal. At a median follow-up of 118 months, 32 women had late DR. CTS5 was a significant prognostic factor for late DR in both pre- and postmenopausal women. In the low CTS5 group, high Ki-67 LI (> 20%) was a significant risk factor for late DR. CTS5 is a useful tool for assessing the risk of late DR in pre- and postmenopausal women with ER-positive breast cancer. Extended endocrine therapy can be considered in patients with high Ki-67 LI (> 20%) in the low CTS5 group.

## Introduction

Over the past few decades, there has been a substantial increase in the incidence of breast cancer worldwide^1–4^. Breast cancer represents a heterogeneous group of diseases, and adjuvant treatments differ among subtypes according to the hormone receptor status^5–7^. Approximately 70–80% of breast cancer patients have an estrogen receptor (ER)-positive subtype, and most are prescribed 5 years of adjuvant endocrine therapy^8,9^. However, in patients with ER-positive breast cancer, the risk of recurrence can extend up to 20 years after completion of 5 years of endocrine treatment^10,11^. Several randomized clinical trials demonstrated that extended adjuvant endocrine treatment could reduce the risk of late recurrence^12–14^.

The Clinical Treatment Score post-5 years (CTS5) is a useful tool based on clinical information that can be used to identify patients who could benefit from extended endocrine therapy beyond 5 years^15^. The CTS5 algorithm was developed to classify the risk of distant recurrence (DR) after 5 years, using two prospective cohorts: ATAC (Arimidex, Tamoxifen, Alone or in Combination) and the BIG (the Breast International Group) 1-98 trial^16,17^. The algorithm was constructed mainly using clinical and pathologic factors including tumor size, number of nodal metastases, grade of the tumor, and age of the patients.

The CTS5, which was developed and validated in two prospective trials, can be clinically used as a supplemental tool to identify the need for extended endocrine therapy in patients who are free of DR after the first 5 years of endocrine therapy. Clinicians could consider recommending prolonged endocrine therapy for patients with high CTS5 score and discontinue it in those with low CTS5 score^18^.

However, this algorithm has not been adequately validated in premenopausal women. It also has one further limitation in terms of the expression of the Ki-67 labelling index (LI). This is an index of tumor proliferation and is considered valuable to aid in distinguishing luminal B-like tumors from luminal A-like tumor in patients with ER-positive breast cancer^19,20^, but this is not included in the CTS5 equation.

Hence, we aimed to test the performance of CTS5 in cohorts from two centers comprising of pre- and postmenopausal patients with ER-positive breast cancer free of DR at 5 years. We then investigated the clinicopathologic parameters including Ki-67 LI to identify factors affecting late recurrence in the CTS5 risk stratification groups.

## Results

### Baseline characteristics

We recruited 680 patients who were followed for a median postoperative period of 118 months. All patients were free of DR during the first 5 years after surgery. Of these, 379 (55.7%) were premenopausal at diagnosis and 301 (44.3%) patients were postmenopausal. The patients’ baseline demographics are summarized in Table 1. The median age at diagnosis was 50.3 years; it was 43.5 years in premenopausal patients and 58.8 years in postmenopausal patients (*P* < .001). The median primary tumor size was 15.0 mm, and 463 (68.1%) patients had pathologically negative nodal status. More than half of the patients (50.3%) had an intermediate tumor grade while 77 (11.3%) patients had a high tumor grade. In the premenopausal group, the proportion of patients with low tumor grade was relatively low; in the postmenopausal group, the proportion of patients with intermediate tumor grade was higher (*P* = .002). There were more patients with progesterone receptor (PR)-positive tumor in the premenopausal group than in the postmenopausal group. A total of 79 (11.6%) patients had human epidermal growth factor receptor 2 (HER2)-positive status. All clinicopathologic characteristics between the pre- and postmenopausal patients were similar except for age, tumor grade, and PR status. Most patients (91.6%) received endocrine treatment for 5 years while 402 (59.1%) patients received chemotherapy (Supplementary Table S1). Premenopausal women were more likely to receive chemotherapy than postmenopausal women (64.6% vs. 52.2%, *P* = .001). Twenty-six (32.9%) HER2-positive patients received adjuvant trastuzumab. Tumor size, number of metastatic lymph nodes, and tumor grade were well classified using the CTS5 algorithm into CTS5 risk groups, but age was not (Supplementary Table S2).

**Table 1:** Patients’ baseline characteristics.

	All patients (%)	Premenopausal patients (%)	Postmenopausal patients (%)	*P* value
Total	680 (100.0)	379 (55.7)	301 (44.3)	
Age at diagnosis, median (range), years	50.3 (25–81)	43.5 (25–55)	58.8 (43–81)	<.001
**Tumor size, mm**				.222
< 10	121 (17.8)	76 (20.1)	45 (15.0)	
10–20	367 (54.0)	198 (52.2)	169 (56.1)	
> 20	192 (28.2)	105 (27.7)	87 (28.9)	
**Pathologic nodal status**				.736
None	463 (68.1)	265 (69.9)	198 (65.8)	
1	107 (15.7)	55 (14.5)	52 (17.3)	
2–3	62 (9.1)	35 (9.2)	27 (9.0)	
4–9	35 (5.1)	18 (4.7)	17 (5.6)	
> 9	13 (1.9)	6 (1.6)	7 (2.3)	
**Histologic grade**				.002
Low	261 (38.4)	161 (42.5)	100 (33.2)	
Intermediate	342 (50.3)	168 (44.3)	174 (57.8)	
High	77 (11.3)	50 (13.2)	27 (9.0)	
**PR**				< .001
Negative	99 (14.6)	30 (7.9)	69 (22.9)	
Positive	581 (85.4)	349 (92.1)	232 (77.1)	
**HER2**				.572
Negative	552 (81.2)	313 (82.6)	239 (79.4)	
Positive	79 (11.6)	41 (10.8)	38 (12.6)	
Unknown	49 (7.2)	25 (6.6)	24 (8.0)	
**Ki-67 LI**				.250
≤ 20%	562 (82.6)	308 (81.3)	254 (84.4)	
> 20%	97 (14.3)	61 (16.1)	36 (12.0)	
Unknown	21 (3.1)	10 (2.6)	11 (3.7)	

PR, progesterone receptor; HER2, human epidermal growth receptor 2; LI, labelling index.

### Performance of CTS5 in pre- and postmenopausal women

**Figure 1:**
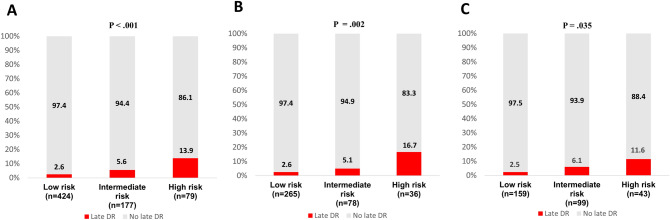
Incidence of late DR by CTS5 subgroups using Chi-square test. (**A**) All patients (*P* < .001); (**B**) Premenopausal women (*P* = .002); (**C**) Postmenopausal women (P = .035). CTS5, Clinical Treatment Score post-5 years; DR, distant recurrence.

Figure 1 summarizes the incidence of late DR according to the CTS5 risk subgroups in pre- and postmenopausal patients. A total of 424 (62.4%) patients were categorized in the low-risk subgroup, 177 (26.0%) in the intermediate-risk subgroup, and 79 (11.6%) in the high-risk subgroup. Among the premenopausal women, 69.9% were at low risk, 20.6% were at intermediate risk, and 9.5% were at high risk. Among the postmenopausal women, 52.8%, 32.9%, and 14.3% were at low, intermediate, and high risk, respectively. During follow-up, 35 events of late DR occurred in 32 (4.7%) patients, 17 in premenopausal and 15 in postmenopausal women. Although lung metastases were more common in the premenopausal group and brain metastases were more common in the postmenopausal patients, there was no statistically significant difference between the groups. The details of the sites of metastasis are summarized in Supplementary Table S3. The group with a high CTS5 score had a higher frequency of late DR (13.9%) compared to the other group (*P* < .001). Similar results were observed in pre and postmenopausal women (*P* = .002 and *P* = .035, respectively).

**Figure 2:**
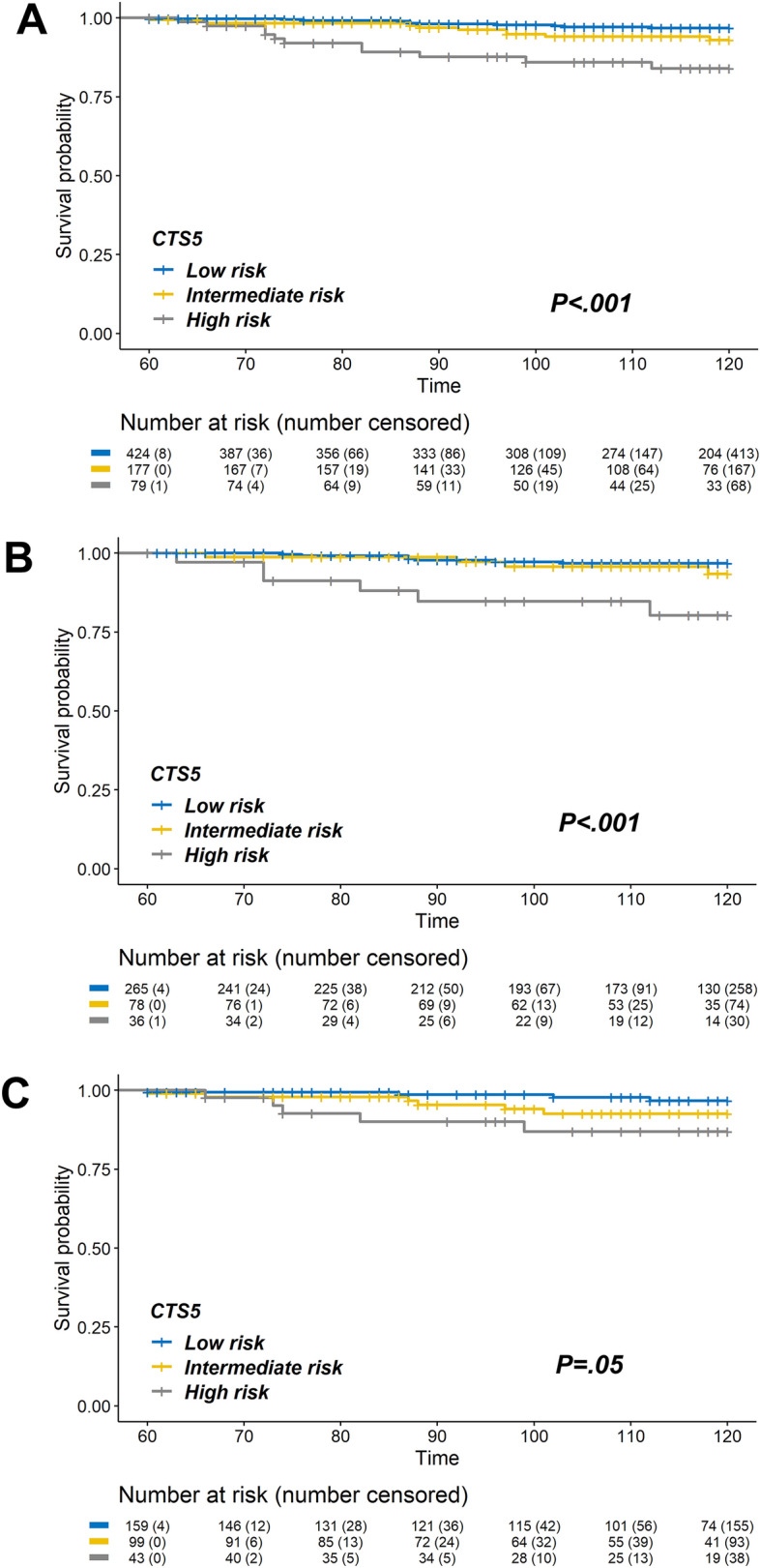
Kaplan–Meier curves for 5–10 years late DR rate according to the CTS5 risk group. (**A**) All patients (log-rank *P* < .001); (**B**) Premenopausal women (log-rank *P* < .001); (**C**) Postmenopausal women (log-rank *P* = .050). CTS5, Clinical Treatment Score post-5 years; DR, distant recurrence.

On Kaplan–Meier analysis, there was a significant difference in the probability of late DR based on the CTS5 category (Fig. 2). Pre- and postmenopausal women in the high-risk group had significantly more DR events (log-rank, *P* < .001 and *P* = .050, respectively).

We evaluated the hazard ratios (HR) of late DR according to the CTS5 score using multivariable Cox regression analysis with adjusted PR, HER2, Ki-67 LI, and chemotherapy (Table 2, Supplementary Table S4-1). CTS5 score as a continuous variable was a significant prognostic factor for late DR in both pre- and postmenopausal women (premenopausal: HR, 3.70; 95% CI 1.66–8.26; *P* = .001; postmenopausal: HR, 3.13; 95% CI 1.53–6.38; *P* = .002) as well as in the entire cohort (HR, 3.23; 95% CI 1.95–5.35; *P* < .001).

**Table 2:** Cox regression analysis of late DR according to risk groups stratified using CTS5.

	Total patients (*N* = 680)		Premenopausal patients (*N* = 379)		Postmenopausal patients (*N* = 301)	
	HR (95% CI)^b^	*P* value	HR (95% CI)^b^	*P* value	HR (95% CI)^b^	*P* value
CTS5 score^a^	3.23 (1.95–5.35)	< .001	3.70 (1.66–8.26)	.001	3.13 (1.53–6.38)	.002
**CTS score**						
Low	Ref		Ref		Ref	
Intermediate	1.99 (0.71–5.60)	.193	1.07 (0.24–4.66)	.934	3.33 (0.77–14.30)	.126
High	6.04 (2.08–17.57)	.001	5.14 (1.40–18.92)	.014	7.52 (1.43–39.67)	.017

CTS5, Clinical Treatment Score post-5 years; DR, distant recurrence; HER2, human epidermal growth factor receptor 2; HR, hazard ratio; LI, labeling index.

^a^Continuous variable.

^b^Adjusted for PR, HER2, Ki-67 LI, chemotherapy.

When patients were divided into three groups according to the CTS5 scores, (Table 2, Supplementary Table S4-2), the group with high scores also had the highest risk of late DR in multivariable analysis. (HR, 6.04; 95% CI 2.08–17.57; *P* = .001). Pre- and postmenopausal patients in the high-risk group also had a high risk of late DR (premenopausal: HR, 5.14; 95% CI 1.40–18.92; *P* = .014, postmenopausal: HR, 7.52; 95% CI 1.43–39.67; *P* = .017). However, the intermediate-risk group did not have a higher risk of late DR compared to the low-risk group (all: HR, 1.99; 95% CI 0.71–5.60; *P* = .193; premenopausal: HR, 1.07; 95% CI 0.24–4.66; *P* = .934; postmenopausal: HR, 3.33; 95% CI 0.77–14.30; *P* = .126).

We performed additional validation in the subgroup with ER-positive/HER2-negative tumors (Table 3). In this cohort as well, CTS5 score as a continuous variable predicted late DR in the entire cohort and in pre- and postmenopausal patients (all: HR, 3.56; 95% CI 2.20–5.77; *P* < .001; premenopausal: HR, 5.51, 95% CI 2.66–11.42; *P* < .001; postmenopausal: HR, 3.41, 95% CI 1.79–6.49; *P* < .001). On comparison between the different risk groups, the risk of late DR in the high CTS5 group was still significantly higher (all: HR, 7.86; 95% CI 2.78–22.24; *P* < .001; premenopausal: HR, 8.64, 95% CI 2.43–30.73, *P* = .001; postmenopausal: HR, 10.06; 95% CI 2.18–46.48, *P* = .003). Furthermore, in the postmenopausal group, patients in the intermediate-risk group had a higher risk of late DR than those in the low-risk group (HR, 5.18; 95% CI 1.36–19.71; *P* = .016).

**Table 3:** Cox regression analysis of late DR according to risk groups by CTS5 in HER2-negative patients.

	All patients (*N* = 552)		Premenopausal patients (*N* = 313)		Postmenopausal patients (*N* = 239)	
	HR (95% CI)^b^	*P* value	HR (95% CI)^b^	*P* value	HR (95% CI)^b^	*P* value
CTS5 score^a^	3.56 (2.20–5.77)	< .001	5.51 (2.66–11.42)	< .001	3.41 (1.79–6.49)	< .001
**CTS score**						
Low	Ref		Ref		Ref	
Intermediate	2.64 (0.98–7.13)	.056	1.93 (0.49–7.59)	.346	5.18 (1.36–19.71)	.016
High	7.86 (2.78–22.24)	< .001	8.64 (2.43–30.73)	.001	10.06 (2.18–46.48)	.003

CTS5, Clinical Treatment Score post-5 years; DR, distant recurrence; HER2, human epidermal growth factor receptor 2; HR, hazard ratio; LI, labeling index.

^a^Continuous variable.

^b^Adjusted for PR, Ki-67 LI, chemotherapy.

### Comparison of observed and expected number of late DR

**Figure 3:**
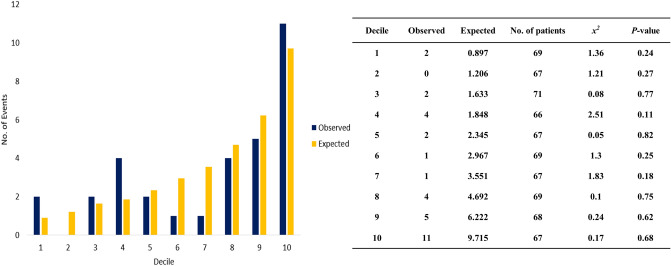
Observed versus expected number of late DR and chi-square values according to the deciles of CTS5. None of the chi-square values were statistically significant. CTS5, Clinical Treatment Score post-5 years; DR, distant recurrence.

In our data, we compared the number of actual occurrences of late DR with those expected based on the CTS5 score (Fig. 3). Based on deciles of the CTS5 score, there was no statistically significant difference between the actual and expected number of late DR in all intervals (Chi-square ≤ 3.84, *P* ≥ .005). Similar results also were observed in pre- and postmenopausal women (Supplementary Figure S1).

### Risk factors of late DR in risk-stratified group

**Figure 4:**
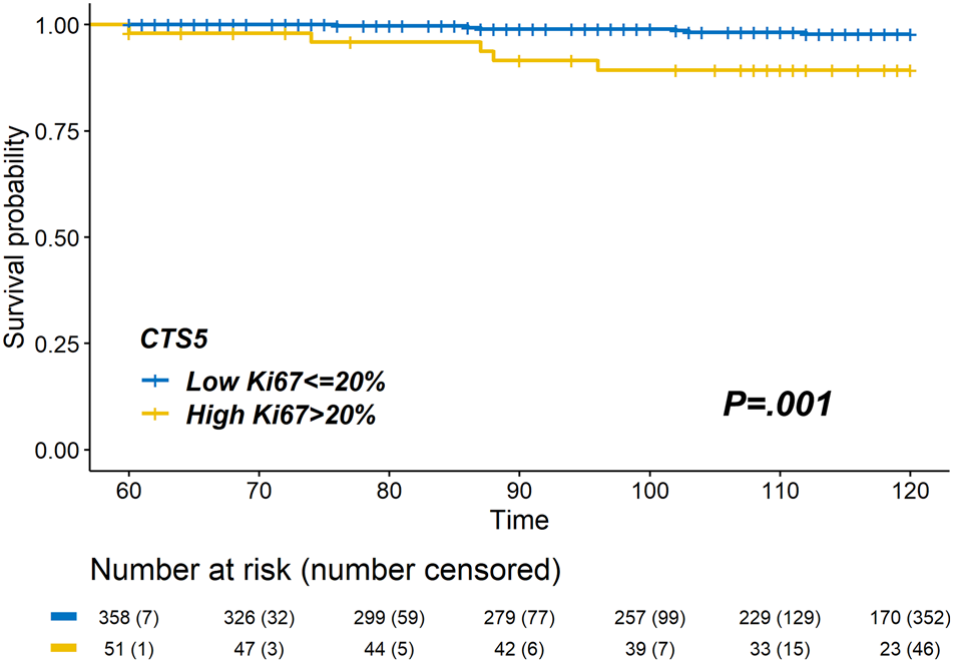
Kaplan–Meier curves for 5–10 years late DR rate according to Ki-67 LI in the CTS5 low-risk group. Late DR rate in patients with high Ki-67 LI was higher than that in the patients with low ki-67 LI in the low CTS5 group (log-rank *P* = .001). CTS5, Clinical Treatment Score post-5 years; DR, distant recurrence; LI, labelling index.

We performed further analyses to identify risk factors of late DR in each CTS5-based risk stratification group. Of all patients, 424 women (62.4%) were categorized into the low-risk group; of these, 11 (2.6%) had late DR. On multivariable Cox regression analysis (Table 4), a higher Ki-67 LI (> 20%) was the only significant factor for late DR in the low-risk group (HR, 5.23; 95% CI 1.46–18.74; *P* = .011). Kaplan–Meier analysis showed that patients with high Ki-67 LI had a higher number of late DR events in the low-CTS5 group. (Fig. 4; log-rank, *P* = .001). However, in the intermediate- and high-risk groups, Ki-67 LI was not associated with late DR. In addition, late DR was associated with high grade tumor and PR-positive status in the high-risk group (Supplementary Table S4).

**Table 4:** Multivariable analysis for late DR in the low risk group by CTS5.

	Low risk patients (*N* = 480)	
	HR (95% CI)	*P* value
Age at diagnosis	0.95 (0.87–1.03)	.163
**Tumor size (mm)**		
< 10	Ref	
10–20	1.95 (0.38–10.15)	.426
> 20	2.95 (0.20–42.60)	.428
**Pathologic nodal status**		
Negative	Ref	
Positive	1.48 (0.15–14.84)	.741
**Histologic grade**		
Low/Intermediate	Ref	
High	0.89 (0.11–7.55)	.915
**Chemotherapy**		
Not administered	Ref	
Administered	0.58 (0.14–2.45)	.461
**Ki-67 LI**		
≤ 20%	Ref	
> 20%	5.23 (1.46–18.74)	.011

HRs of PR, HER2 could not be estimated.

CTS5, Clinical Treatment Score post-5 years; DR, distant recurrence; HER2, human epidermal growth factor receptor 2; HR, hazard ratio; LI, labelling index.

## Discussion

Our study revealed that CTS5 is useful in estimating the risk of late DR among pre- and postmenopausal patients with ER-positive breast cancer who are free of DR until 5 years after surgery. Although the algorithm was developed based on cohorts comprising postmenopausal women at diagnosis, CTS5 predicted the risk of late DR in premenopausal women as well. Another recent study to evaluate CTS5 in non-ATAC and BIG 1-98 trial patients including premenopausal women also suggested the validity of the CTS5 for predicting late DR in premenopausal women^21^. These findings support the use of CTS5 score for estimating the risk of late DR in premenopausal women.

Furthermore, we found that high Ki-67 LI was associated with late DR in the low CTS5 group. The actual incidence of late DR was low (2.5–3.6%) in the low CTS5 group; however, it is uncomfortable if late DR occurs after the end of endocrine therapy despite a low CTS5 score. Hence, it is important to identify risk factors of late DR in the low CTS5 group. The late DR rate in patients with a high Ki-67 LI (> 20%) in the low-risk group was 9.8%, which was higher than that in the intermediate CTS5 group. Our results suggest that clinicians should be aware of the potential benefit of extended endocrine therapy after 5 years in a patient with a high Ki-67 LI (> 20%) and a low CTS5 score.

Ki-67 LI is a representative biomarker for tumor proliferation and has been reported to be associated with early recurrence in ER-positive breast cancer^22,23^. Regarding risk factors for late recurrence, numerous studies suggested that the tumor burden, size, and nodal involvement are most important factors rather than the proliferation index. Currently, biomarkers reflecting proliferation including Ki-67 LI have not been extensively studied in the context of late recurrence in ER-positive breast cancer. However, some studies showed that proliferating markers are associated with late recurrence in ER-positive breast cancer. Bianchini et al. showed that tumors with highly proliferative/high ER-related scores had the highest relapse rates after 5 years of adjuvant tamoxifen therapy^24^. Conforti et al. also reported that Ki-67 LI was associated with late DR in ER-positive breast cancer and that late DR could be predicted more accurately by combining CTS5 and Ki-67 LI, although their study included only lobular carcinoma^25^. These studies support our results that some tumors with high Ki-67 LI could relapse despite a low CTS5 score. Although we could not demonstrate that extended endocrine therapy will be helpful in a highly proliferative case, we can assume that extension of endocrine therapy might offer a clinical benefit in reducing late DR for the low CTS5 tumors with high Ki-67 LI as many consider extended endocrine therapy in the high CTS5 group because they have more chance of late DR. Considered together, extended endocrine therapy could be a possibility in patients with low CTS5 but high Ki-67 LI due to the higher risk of late DR.

In this study, 62.4%, 26.0%, and 11.6% of patients were in the low-, intermediate-, and high-risk groups, respectively, which was different from the proportions reported in the ATAC and BIG 1-98 trials. In the ATAC cohort, 42.0%, 31.3%, and 20.7% of the patients were in the low-, intermediate-, and high-risk groups respectively, while in the BIG 1-98 cohort 42.6%, 31.8%, and 25.5% patients were in the low-, intermediate-, and high-risk groups respectively. These differences might be due to the differences in tumor grade. In our study, only 11.3% of the patients had high-grade tumor. In contrast, 25.3% of patients in the ATAC cohort and 20.3% of patients in the BIG 1-98 cohort had high-grade tumor.

The CTS5 algorithm can be carefully used for patients with ER-positive/HER2-positive breast cancer, because it has not been validated in such patients. After the ATAC and BIG 1-98 trials, trastuzumab was introduced for patients with HER2-positive disease^18,19^. In our study, 11.6% of the patients had HER2-positive tumors, and trastuzumab treatment was administered to 32.9% of these patients. Further validation is warranted among patients with the ER-positive/HER2-positive subtype so as to document the accuracy of CTS5 score in these patients.

In patients belonging to the intermediate CTS5 score group, Ki-67 LI was not identified as a risk factor for late DR; thus, decision-making for extension of endocrine therapy remains difficult. Until now, it might be best for the clinicians to decide extended endocrine therapy after discussion with the patients by considering clinicopathologic risk factors as well as potential benefit and toxicity of extended treatment. Since the late DR rate of intermediate CTS5 group was 5.6% in our cohort, and no statistically difference from the late DR rate of low CTS5 group, we thought that extension of endocrine therapy was carefully omitted in these patients. However, more validation studies in large cohorts are necessary in order to obtain clear conclusion.

Richman et al. suggested that genomic assays such as PAM50 or Breast Cancer Index could be integrated into the clinical-genomic stratification of the risk of late DR^18^. Particularly, in the patients with an intermediate CTS5 score, they suggested discontinuation of endocrine therapy for patients with a low genomic score or recommendation of extended endocrine therapy for up to 10 years in patients with a high genomic score. Finally, they recommended that women with an intermediate score should discuss the probability of toxicity and their own personal preferences with the clinicians. This clinical scenario with the use of genomic assay for the intermediate CTS5 group needs to be supported by further evidence and prospective studies.

Moreover, in the era of widespread use of genomic assays in ER-positive disease, many investigators postulate whether CTS5 could be integrated or combined with genomic tools to determine the need for extended endocrine therapy. A recent validation of the CTS5 tool in the TAILOR-X study showed that CTS5 is strongly prognostic for those who were deemed as intermediate- or high-risk by Oncotype Dx RS (11–100)^26^. Since the TAILOR-X study was not designed to compare the effect of extended endocrine therapy, it remains unclear how clinicians could integrate genomic testing with CTS5 score in decision-making for extension of endocrine therapy.

The limitations of this study include selection bias due to its retrospective design. Despite the fact that the prognosis of patients with ER-positive breast cancer was favorable, it should be noted that our sample size was small. Furthermore, among premenopausal women in our study, only 103 (15.1%) received ovarian function suppression (OFS) therapy within 5 years. A new validation study on CTS5 comprising a cohort of premenopausal women treated with OFS is warranted in the future. In addition, we could not validate whether CTS5 is reliable in ER-positive/HER2-positive patients. Validation of CTS5 in an ER-positive cohort, including in HER2-positive patients treated homogenously with anti-HER2 therapy, is necessary. Nevertheless, since we analyzed the data from two institutions, we could obtain more comprehensive information such as variable clinicopathologic parameters in a homogeneous cohort. Although our results are based on a retrospective analysis, they are consistent with those of the ATAC and BIG 1-98 trials for stratifying patients into risk categories for late DR.

In conclusion, CTS5 is a useful tool for ER-positive breast cancer patients at risk for late DR among pre- and postmenopausal women. Extended endocrine therapy could be also considered for patients with high Ki-67 LI (> 20%) in the low CTS5 group, because high Ki-67 LI is a risk factor associated with late DR for patients with a low CTS5 score.

## Methods

### Study populations

From the Gangnam Severance Hospital and Severance Hospital breast cancer registry, we retrospectively identified patients with ER-positive primary breast cancer. The database registry includes data on clinicopathologic information and survival outcomes. All patients underwent curative surgery for primary breast cancer between January 2004 and December 2009 at Gangnam Severance Hospital and between April 2008 and December 2009 at Severance Hospital. Patients who received neoadjuvant treatment or had de novo stage IV disease were excluded, because the CTS5 score requires knowledge of the accurate number of involved nodes. Patients who received extended endocrine therapy were also excluded as CTS5 predicts the need for extension of endocrine therapy. The study was conducted in accordance with the Good Clinical Practice guidelines and the tenets of the Declaration of Helsinki. The method was approved by the institutional review board (IRB) of Gangnam Severance Hospital. The IRB of Gangnam Severance Hospital granted a waiver of written documentation of informed consent from all participants because of the retrospective study design.

Among ER-positive patients who received endocrine treatment, 680 patients who were free of DR for 5 years after surgery were included in this study. All patients underwent abdominal/chest computed tomography and whole-body bone scan during the annual follow-up. We obtained the following clinical and pathological variables: age at diagnosis, tumor size (in millimeters), nodal status (negative, positive), histologic grade (low, intermediate, high), PR status (negative, positive), and HER2 status (negative, positive). The Ki-67 LI was averaged at three representative areas on the invasive side of the tumor and presented as a percentage score (range 0–100%) of positive tumor cells. This was used to divide the patients into two groups (those with > 20% positive tumor cells and those with less).

### Immunohistochemistry study

We evaluated ER, PR, HER2, and Ki-67 LI status using 1:100 clone 6F11 (Novocastra, Newcastle upon Tyne, UK), clone 16 (Novocastra), 4B5 rabbit monoclonal antibody (Ventana Medical Systems, Tucson, AZ, USA), and MIB-1 (Dako, Glostrup, Denmark) antibodies, respectively. ER- and PR-positive immunohistochemical expressions were evaluated according to the modified Allred system^27^. HER2 status was determined according to the American Society of Clinical Oncology/College of American Pathologists guideline^28^. Ki-67 LI expression was reported as percentage (range, 0–100%) of positive tumor cells and dichotomized using a cut-off of 20%^29^.

### Definition of menopause

A questionnaire on menopause was administered during the first diagnosis. Patients with regular/irregular vaginal bleeding within the last year were defined as premenopausal. Perimenopausal women, whose last menstrual cycle was between the last 1 year and last 1 month, were also classified as premenopausal. If the menopausal status was unknown due to past history of hysterectomy or intrauterine device (IUD) insertion, we referred to the follicular stimulating hormone (FSH) levels from the blood test. Patients with FSH levels below 30 mIU/mL were considered premenopausal.

### CTS5 score

The final CTS5 algorithm with coefficients based on ATAC and BIG 1-98 datasets was used (https://www.cts5-calculator.com). The variables used were patient age, tumor size, nodal status, and tumor grade^15^:

$${\text{CTS5 }} = \, 0.{438} \times {\text{number of involved nodes }} + \, 0.{988} \times (0.0{93} \times {\text{size }} - \, 0.00{1} \times {\text{size}}^{{2}} + \, 0.{375} \times {\text{grade }} + \, 0.0{17} \times {\text{age}})$$

Patients were categorized into the risk stratification groups according to the cut-off points for CTS5 as low (CTS5 < 3.13), intermediate (3.13–3.86), and high (> 3.86) risk.

### Statistical analysis

The primary objective of this study was to determine whether CTS5 can predict the risk of late DR (5–10 years postoperative) in pre- and postmenopausal women. Late DR was defined as metastasis between 5 and 10 years after surgery, excluding contralateral disease and locoregional or ipsilateral recurrences. Kaplan–Meier survival estimates with corresponding 95% confidence intervals (CI) were used to determine the prognostic performance of CTS5, while comparisons of survival curve between the risk subgroups were investigated using the log-rank test. Clinicopathologic factors were compared using the chi-square test for categorical variables. Continuous variables were compared using the independent two-sample t-test. A Cox proportional hazard model was used to identify variables associated with late DR. To compare the observed and expected values, the chi-square test was used in each decile of the expected risk by CTS5. *P* values < .05 were considered statistically significant. SPSS version 25.0 (IBM Inc., Armonk, NY, USA) and an R package, version 3.6.1 (https://www.R-project.org) were used for the statistical analyses.

## Supplementary information

Supplementary Figure Legend.

Supplementary Figure S1.

Supplementary Table S1.

## Data Availability

The datasets used and/or analyzed during the current study are available from the corresponding author on reasonable request.
