# Author Correction: Validation of Clinical Treatment Score post-5 years (CTS5) risk stratification in premenopausal breast cancer patients and Ki-67 labelling index

**DOI:** 10.1038/s41598-021-84773-x

**Published:** 2021-03-08

**Authors:** Janghee Lee, Chihwan Cha, Sung Gwe Ahn, Dooreh Kim, Soeun Park, Soong June Bae, Jeeye Kim, Hyung Seok Park, Seho Park, Seung Il Kim, Byeong‑Woo Park, Joon Jeong

**Affiliations:** 1 Department of Surgery, Gangnam Severance Hospital, Yonsei University College of Medicine, 712 Eonjuro, Gangnam‑gu, Seoul, 06273 Republic of Korea; 2 Department of Surgery, Severance Hospital, Yonsei University College of Medicine, Seoul, Republic of Korea; 3 Department of Surgery, Sacred Heart Hospital, Hallym University, Dongtan, Republic of Korea; 4 Department of Surgery, Hanyang University College of Medicine, Seoul, Republic of Korea

Correction to: *Scientific Reports* 10.1038/s41598-020-74055-3, published online 08 October 2020

The Acknowledgements section in this Article is incomplete.

“We would like to thank Editage (https://www.editage.co.kr) for English language editing.”

should read:

“This research was supported by the Basic Science Research Program through the NRF, funded by the Ministry of Science, ICT & Future Planning (NRF-2019R1C1C1002830); a grant from the National R&D Program for Cancer Control, Ministry of Health & Welfare, Republic of Korea (1520120). We would like to thank Editage (https://www.editage.co.kr) for English language editing.”

