# Supplementary material for: Validation of Clinical Treatment Score post-5 years (CTS5) risk stratification in premenopausal breast cancer patients and Ki-67 labelling index
Source: Sci Rep. 2020 Oct 8;10:16850. doi: 10.1038/s41598-020-74055-3 (PMC7546620; doi:10.1038/s41598-020-74055-3)
Supplement: Supplementary file 1 — Supplementary Figure Legend. [file 41598_2020_74055_MOESM1_ESM.docx]

**Supplementary Figure S1.** Observed versus expected number of late DR and chi-square value according to deciles of CTS5 in pre- and postmenopausal women. (A) Premenopausal women (B) Postmenopausal women; Most of the chi-square were not statistically significant except 1 interval in premenopausal women.

DR, distant recurrence; CTS5, Clinical Treatment Score post-5 years.
