# Supplementary figures and images for: Validation of Clinical Treatment Score post-5 years (CTS5) risk stratification in premenopausal breast cancer patients and Ki-67 labelling index
Source: Sci Rep. 2020 Oct 8;10:16850. doi: 10.1038/s41598-020-74055-3 (PMC7546620; doi:10.1038/s41598-020-74055-3)

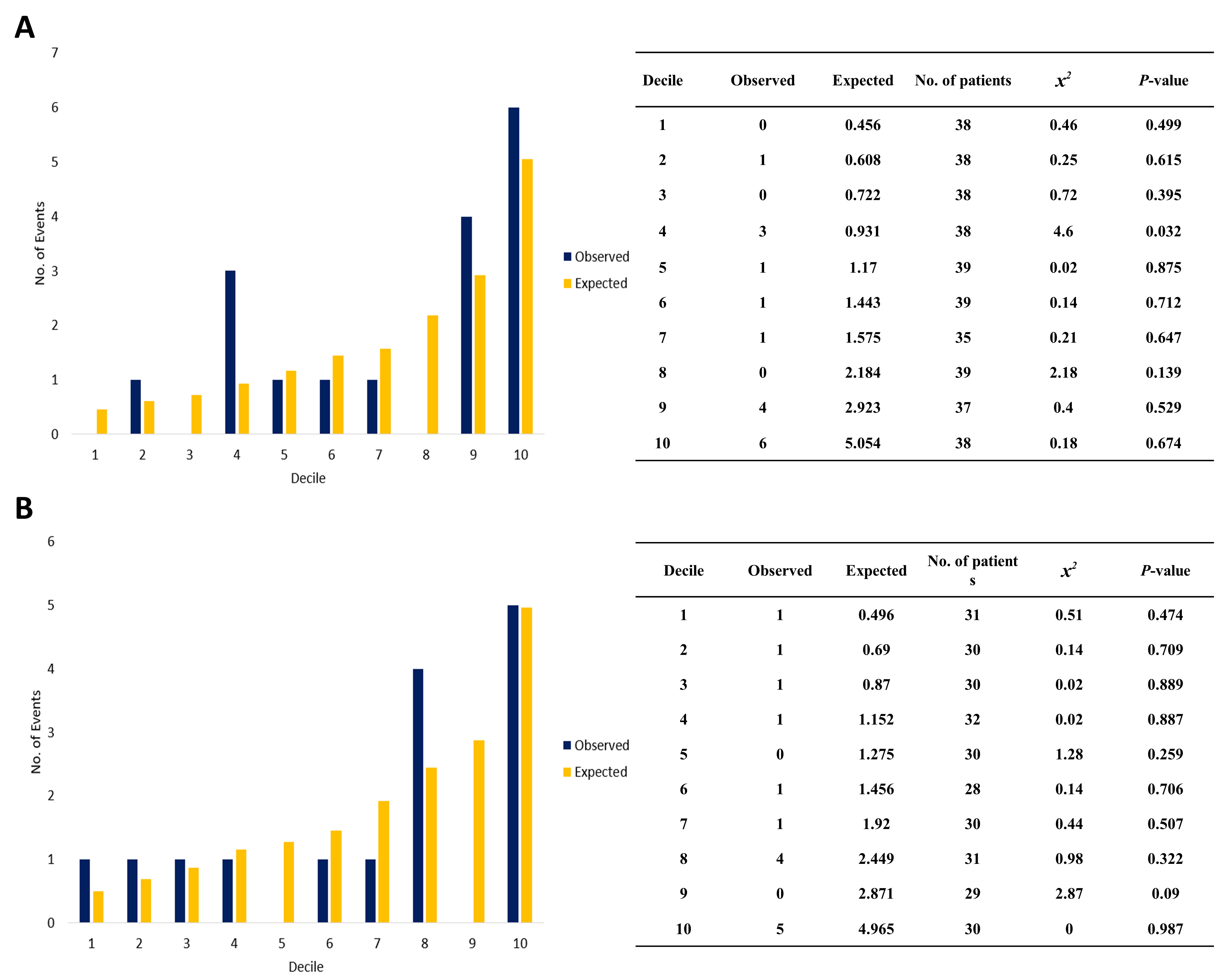

Supplement: Supplementary file 2 — Supplementary Figure S1. [file 41598_2020_74055_MOESM2_ESM.tif]
