# Supplementary material for: Validation of Clinical Treatment Score post-5 years (CTS5) risk stratification in premenopausal breast cancer patients and Ki-67 labelling index
Source: Sci Rep. 2020 Oct 8;10:16850. doi: 10.1038/s41598-020-74055-3 (PMC7546620; doi:10.1038/s41598-020-74055-3)
Supplement: Supplementary file 3 — Supplementary Table S1. [file 41598_2020_74055_MOESM3_ESM.pdf]

Validation of Clinical Treatment Score post-5 years (CTS5) risk stratification in premenopausal breast cancer patients and Ki-67 labelling index

Janghee Lee[1,3†], Chihwan Cha[4†], Sung Gwe Ahn[1*], Dooreh Kim[1], Soeun Park[1], Soong June Bae[1], Jeeye Kim[2], Hyung Seok Park[2], Seho Park[2], Seung Il Kim[2], Byeong-Woo. Park[2] and Joon Jeong[1*]

[1]Department of Surgery, Gangnam Severance Hospital, Yonsei University College of Medicine, Seoul, Republic of Korea

[2]Department of Surgery, Severance Hospital, Yonsei University College of Medicine, Seoul, Republic of Korea

[3]Department of Surgery, Sacred Heart Hospital, Hallym University, Dongtan, Republic of Korea

[4]Department of Surgery, Hanyang University College of Medicine, Seoul, Republic of Korea

[†]These authors contributed equally to this work.

[*]**Co-Correspondence to:** Joon Jeong, MD, PhD

Department of Surgery, Gangnam Severance Hospital, 712 Eonjuro, Gangnam-gu, Seoul, 06273, Republic of Korea. Tel: +82-2-2019-3370. E-mail: gsjjoon@yuhs.ac

[*]**Co-Correspondence to:** Sung Gwe Ahn, MD, PhD

Department of Surgery, Gangnam Severance Hospital, 712 Eonjuro, Gangnam-gu, Seoul, 06273, Republic of Korea. Tel: +82-2-2019-3370. E-mail: asg2004@yuhs.ac

Supplementary Table S1. Surgery and adjuvant treatment of patients

	All patients (%)	Premenopausal patients (%)	Postmenopausal patients (%)	*P* value
Total	680 (100.0)	379 (55.7)	301 (44.3)	
Type of Surgery				.388
Total mastectomy	376 (55.3)	204 (53.8)	172 (57.1)	
Partial mastectomy	304 (44.7)	175 (46.2)	129 (42.9)	
Radiotherapy				.717
Not administered	311 (45.7)	171 (45.1)	140 (46.5)	
Administered	369 (54.3)	208 (54.9)	161 (53.5)	
Chemotherapy				.001
Not administered	278 (40.9)	134 (35.4)	144 (47.8)	.
Administered	402 (59.1)	245 (64.6)	157 (52.2)	
Endocrine Therapy				.830
Not complete 5yr	57 (8.4)	31 (8.2)	26 (8.6)	
Complete 5 yr	623 (91.6)	348 (91.8)	275 (91.4)	
SERM	325 (52.2)	301 (86.5)	24 (8.7)	
AI	260 (41.7)	12 (3.4)	248 (90.2)	
Switch	38 (6.1)	35 (10.1)	3 (1.1)	
Trastzumab use in HER2+				.474

Not administered	53 (67.1)	29 (70.7)	24 (63.2)
Administered	26 (32.9)	12 (29.3)	14 (36.8)

SERM, selective estrogen receptor modifier; AI, aromatase inhibitor; HER2, human epidermal growth receptor 2

Supplementary Table S2. Patients' clinicopathologic feature according to risk groups stratified using CTS5

	Low risk group (%, *N* = 424)	Intermediate risk group (%, *N* = 177)	High risk group (%, *N* = 79)	*P* value
Age				.110
<50	228 (53.8)	79 (44.6)	38 (48.1)	
≥50	196 (46.2)	98 (55.4)	41 (51.9)	
Tumour size, mm				<.001
<10	119 (28.1)	2 (1.1)	0 (0.0)	
10-20	270 (63.7)	73 (41.2)	24 (30.4)	
>20	35 (8.3)	102 (57.6)	55 (69.6)	
Pathologic nodal status				<.001
None	383 (90.3)	79 (44.6)	1 (1.3)	
1	35 (8.3)	61 (34.5)	11 (13.9)	
2-3	6 (1.4)	32 (18.1)	24 (30.4)	
4-9	0 (0.0)	4 (2.3)	31 (39.2)	
> 9	0 (0.0)	1 (0.6)	12 (15.2)	
Histologic grade				<.001
Low	211 (49.8)	35 (19.8)	15 (19.0)	
Intermediate	187 (44.1)	108 (61.0)	47 (59.5)	
High	26 (6.1)	34 (19.2)	17 (21.5)	

PR				.822
Negative	59 (13.9)	28 (15.8)	12 (15.2)	
Positive	365 (86.1)	149 (84.2)	67 (84.8)	
HER2				<.001
Negative	358 (84.3)	135 (76.3)	59 (74.7)	
Positive	31 (7.3)	34 (19.2)	14 (17.7)	
Unknown	35 (8.3)	8 (4.5)	6 (7.6)	
Ki-67 LI				.254
≤20%	358 (84.4)	140 (79.1)	64 (81.0)	
>20%	51 (12.0)	33 (18.6)	13 (16.5)	
Unknown	15 (3.5)	4 (2.3)	2 (2.5)	

CTS5, Clinical Treatment Score post-5 years; PR, progesterone receptor; HER2, human epidermal growth receptor 2; LI, labelling index

Supplementary Table S3. Distribution of organ with late DR

Organ with late DR	All patients (%, *N*=680)	Premenopausal patients (%, *N*=379)	Postmenopausal patients (%, *N*=301)	*P* value
Total	35 (100.0)	19 (53.1)	16 (46.9)	.761
Bone	12 (100.0)	6 (50.0)	6 (50.0)	.687
Lung	8 (100.0)	6 (75.0)	2 (25.0)	.270
Liver	3 (100.0)	2 (66.7)	1 (33.3)	.702
Brain	7 (100.0)	2 (28.6)	5 (71.4)	.146
Lymph node	4 (100.0)	2 (50.0)	2 (50.0)	.817
Other	1 (100.0)	1 (100.0)	0 (0.0)	.372

DR, distant recurrence

Supplementary Table S4-1. Multivariable Cox regression analysis of late DR according to continuous valuable of CTS5

	All patients (N = 680)		Premenopausal patients (N = 379)		Postmenopausal patients (N = 301)	
	HR (95% CI)	P value	HR (95% CI)	P value	HR (95% CI)	P value
PR						
Negative	Ref.		Ref.		Ref.	
Positive	0.62 (0.25-1.53)	.298	0.72 (0.16-3.20)	.663	0.56 (0.17-1.87)	.349
HER2						
Negative	Ref.		Ref.		Ref.	
Positive	0.33 (0.08-1.42)	.136	0.34 (0.04-2.63)	.302	0.36 (0.04-2.97)	.341
Ki-67 LI						
≤ 20 %	Ref.		Ref.		Ref.	
> 20%	2.37 (1.02-5.49)	.044	2.90 (1.03-8.26)	.045	1.53 (0.32-7.26)	.594
Chemotherapy						
Not administered	Ref.		Ref.		Ref.	

Administered	0.46 (0.18-1.18)	.107	0.52 (0.11-2.56)	.420	0.38 (0.10-1.38)	.140
CTS5 score*	3.23 (1.95-5.35)	<.001	3.70 (1.66-8.26)	.001	3.13 (1.53-6.38)	.002

*continuous variable

DR, distant recurrence; CTS5, Clinical Treatment Score post-5 years; HR, hazard ratio; PR, progesterone receptor; HER2, human epidermal growth receptor 2; LI, labelling index

Supplementary Table S4-2. Multivariable Cox regression analysis of late DR according to risk groups stratified using CTS5

	All patients (N = 680)		Premenopausal patients (N = 379)		Postmenopausal patients (N = 301)	
	HR (95% CI)	P value	HR (95% CI)	P value	HR (95% CI)	P value
PR						
Negative	Ref.		Ref.		Ref.	
Positive	0.57 (0.23-1.41)	.223	0.65 (0.15-2.90)	.570	0.53 (0.16-1.79)	.307
HER2						
Negative	Ref.		Ref.		Ref.	
Positive	0.33 (0.08-1.45)	.143	0.41 (0.05-3.17)	.391	0.33 (0.04-2.85)	.316
Ki-67 LI						
≤ 20 %	Ref.		Ref.		Ref.	
> 20%	2.41 (1.04-5.60)	.041	3.12 (1.09-8.89)	.034	1.66 (0.34-8.15)	.531
Chemotherapy						
Not administered	Ref.		Ref.		Ref.	
Administered	0.67	.432	1.30	.731	0.41	.200

	(0.25-1.82)		(0.30-5.63)		(0.11-1.59)	
CTS5 score						
Low	Ref.		Ref.		Ref.	
Intermediate	1.99	.193	1.07	.934	3.33	.126
	(0.71-5.60)		(0.24-4.66)		(0.77-14.30)	
High	6.04	.001	5.14	.014	7.52	.017
	(2.08-17.57)		(1.40-18.92)		(1.43-39.67)	

DR, distant recurrence; CTS5, Clinical Treatment Score post-5 years; HR, hazard ratio; PR, progesterone receptor; HER2, human epidermal growth receptor 2; LI, labelling index

Supplementary Table S5. Multivariable analysis of late DR in the intermediate- and high risk CTS5 groups

	Intermediate risk patients (N = 177)		High risk patients (N = 79)	
	HR (95% CI)	P value	HR (95% CI)	P value
Age at diagnosis	1.04 (0.96-1.12)	.386	1.01 (0.94-1.09)	.784
Tumor size (mm)				
<10	Ref.		.	
10-20	NE	NE	Ref	
>20	NE	NE	8.01 (0.37-NE)	.185
Pathologic nodal status				
Negative	Ref.		Ref.	
Positive	1.99 (0.30-13.05)	.474	NE	NE
Histologic grade				
Low/Intermediate	Ref.		Ref.	
High	0.80 (0.08-8.43)	.852	5.74 (1.15-28.70)	.033
PR				
Negative	Ref.		Ref.	
Positive	0.49 (0.09-2.75)	.416	0.17 (0.04-0.83)	.029
HER2				

Negative	Ref.		Ref.	
Positive	0.71 (0.08-6.29)	.754	0.43 (0.04-4.41)	.477
Chemotherapy				
Not administered	Ref.		Ref.	
Administered	0.47 (0.07-3.26)	.442	0.13 (0.01-3.10)	.205
Ki-67 LI				
≤ 20 %	Ref.		Ref.	
> 20 %	2.10 (0.39-11.18)	.392	0.37 (0.03-4.08)	.419

DR, distant recurrence; CTS5, Clinical Treatment Score post-5 years; HR, hazard ratio; PR, progesterone receptor; HER2, human epidermal growth receptor 2; LI, labelling index; NE, not estimated
